# *MAP1B* Variants Disrupt Neuronal Migration: Insights From Three Novel Families

**DOI:** 10.1111/cge.70059

**Published:** 2025-08-28

**Authors:** Jessica Archer, Matt Edwards, Thomas Macdougall, Anne Baxter, Himanshu Goel

**Affiliations:** ^1^ Hunter Genetics Waratah New South Wales Australia; ^2^ In Focus Radiology Wallsend New South Wales Australia; ^3^ University of Newcastle Callaghan New South Wales Australia

**Keywords:** cortical malformations, intellectual disability, MAP1B, neurodevelopmental, periventricular nodular heterotopia, polymicrogyria, PVNH, seizures

## Abstract

*MAP1B* (microtubule‐associated protein 1B) encodes a cytoskeletal regulator critical for neuronal migration, axon guidance, and cortical circuit formation. Disease‐causing variants (DCVs) in *MAP1B* have recently emerged as a cause of neurodevelopmental disorders characterized by intellectual disability, epilepsy, and cortical malformations, including periventricular nodular heterotopia (PVNH) and polymicrogyria (PMG). However, the phenotypic and neuroimaging spectrum associated with *MAP1B*‐related disease remains incompletely defined. We describe seven affected individuals from three unrelated families with pathogenic *MAP1B* variants. Clinical, neuroimaging, and genetic data were analyzed in the context of emerging literature to delineate the pathogenic mechanisms and phenotypic variability associated with *MAP1B* dysfunction. All individuals carried loss of function *MAP1B* variants. Clinical features included global developmental delay, intellectual disability, behavioural dysregulation, and focal epilepsy. Neuroimaging revealed anteriorly predominant PVNH in four of five cases with neuroimaging available. These findings reinforce *MAP1B*'s central role in cytoskeletal regulation, neuronal positioning, and synaptic connectivity. Functional data from animal and cell models support a mechanism involving impaired microtubule stabilization, altered growth cone dynamics, and dysregulated axon branching. Our case series expands the clinical and radiological phenotype associated with *MAP1B*‐related disorders and highlights its position as a key cytoskeletal regulator in human corticogenesis. Systematic genotype–phenotype correlation and functional studies are needed to inform diagnostic interpretation and explore therapeutic avenues in *MAP1B*‐associated disease.

## Graphical abstract

Pathogenic variants in *MAP1B* have recently emerged as a cause of neurodevelopmental disorders characterized by intellectual disability, epilepsy, and cortical malformations, including periventricular nodular heterotopia (PVNH) and polymicrogyria (PMG). We describe clinical, neuroimaging, and genetic data from seven affected individuals from three unrelated families with loss‐of‐function *MAP1B* variants.

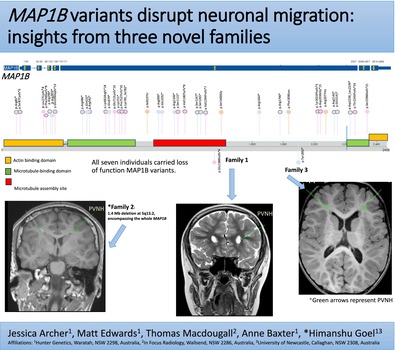

## 1 Introduction

*MAP1B* encodes a high‐molecular‐weight microtubule‐associated protein enriched in the developing brain, where it plays a pivotal role in neuronal migration, axon elongation, cytoskeletal remodelling, and synapse formation. Early studies in murine models identified *MAP1B* as a regulator of both microtubule and actin filament dynamics, with phosphorylation‐dependent modulation by kinases such as GSK3β and DYRK1A finely tuning its function [1]. DCVs in *MAP1B* have recently emerged as contributors to neurodevelopmental disorders, notably cortical malformations such as PVNH and PMG^2^. PVNH is characterised by heterotopic neuronal clusters lining the lateral ventricles, arising from impaired radial migration during embryogenesis [2]. There are many genetic associations of PVNH, although few are recurrent. Although DCVs in *FLNA* are most commonly implicated in PVNH, other cytoskeletal regulators including *ARFGEF2* and *EML1* have also been associated with this phenotype, highlighting the complex molecular architecture governing neuronal migration [3]. *MAP1B*'s functional interaction with these proteins suggests a broader regulatory role within cytoskeletal signalling networks [4].

To further delineate the clinical and imaging spectrum of *MAP1B*‐related disorders, we describe seven individuals from three families harboring pathogenic or likely pathogenic variants in *MAP1B*. These cases exhibited overlapping neurodevelopmental phenotypes, including intellectual disability, epilepsy, and structural brain anomalies, thereby expanding our understanding of *MAP1B*'s contribution to cortical development and highlighting its relevance to diagnosis and prognostication in neurodevelopmental disorders.

## 2 Case Reports

All available brain MRI scans were independently reviewed by a radiologist (TM) with expertise in cortical malformations. The imaging was assessed for periventricular nodular heterotopia, subtle polymicrogyria, corpus callosum anomalies, and other neuromigrational abnormalities. Findings were discussed jointly with the clinical team to reach consensus on the radiological interpretation.

### 2.1 Family 1

**FIGURE 1:**
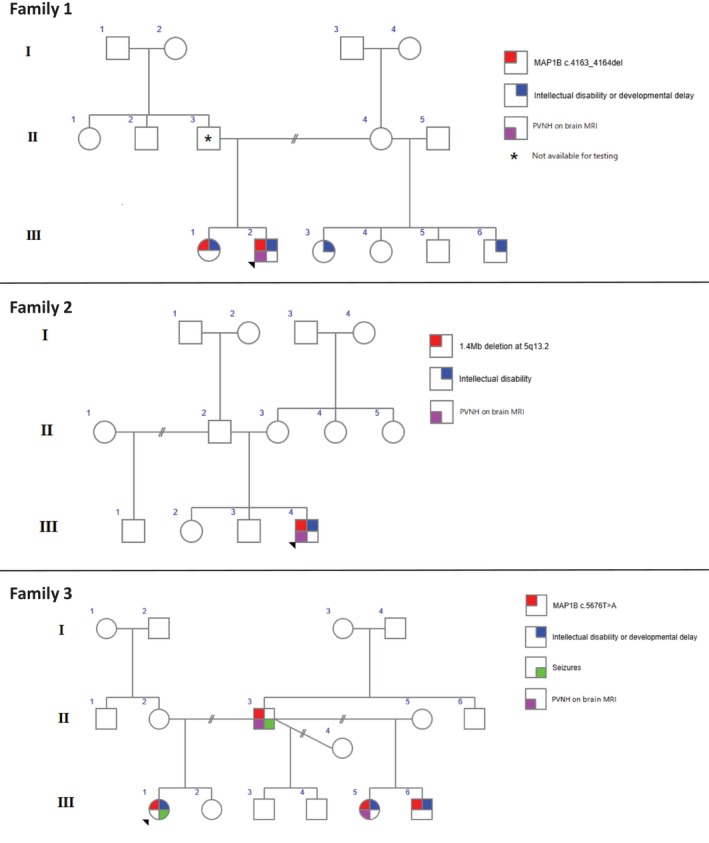
Family pedigrees of the three families presented in this cohort.

**FIGURE 2:**
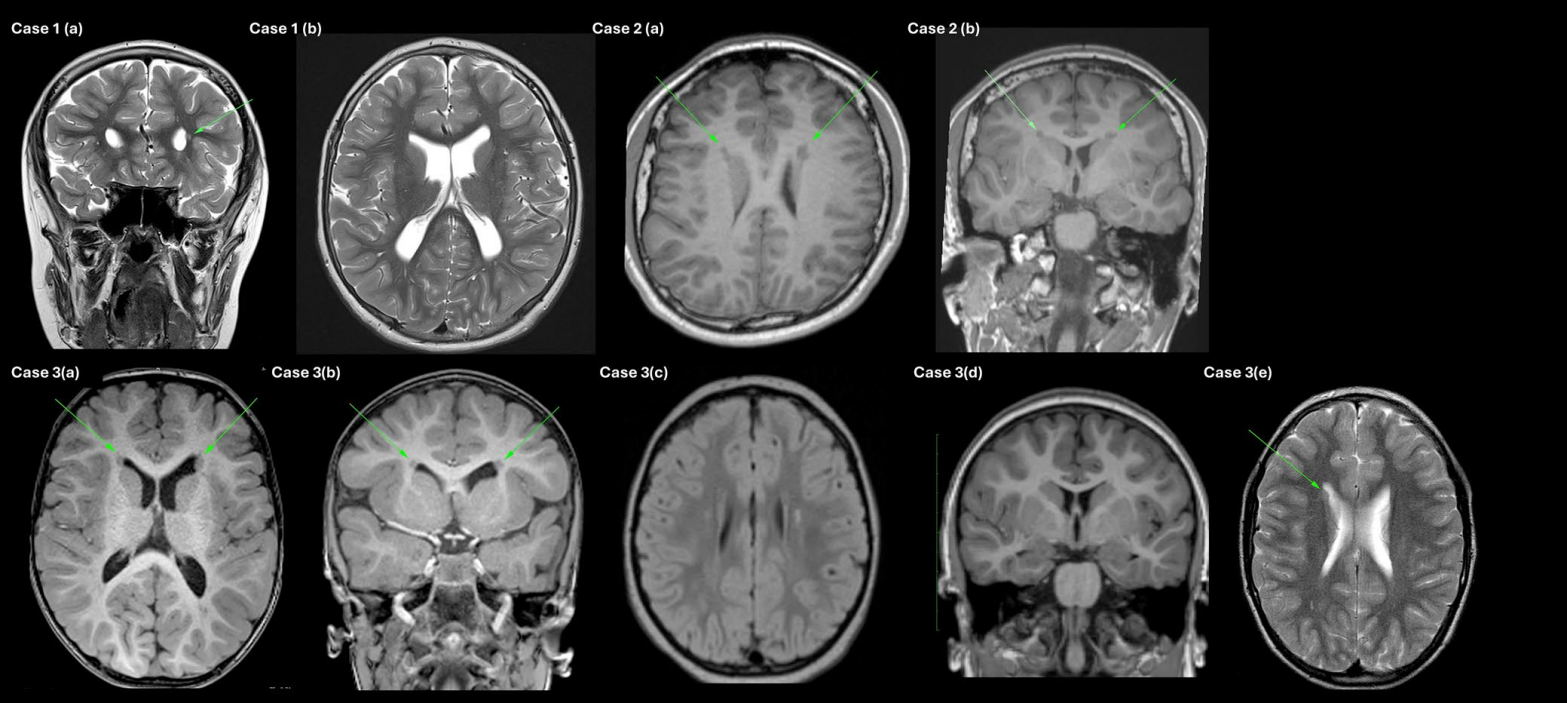
MRI brain images from patients (arrows indicate PVNH). Case 1 coronal (a) and axial (b) T2 weighted images demonstrating PVNH around the left frontal horn and prominent ventricles and perivascular spaces. Case 2 Axial (a) and coronal (b) T1 weighted images, demonstrating PVNH around the frontal horns. Case 3. Axial (a) and coronal (b) T1 weighted images of III‐5 demonstrating PVNH. Axial FLAIR (c) and coronal T1 weighted (d) images of III‐1 showing abnormal T2 high signal in the corona radiata, but no evidence of PVNH. Axial (e) T2 weighted image of the father (II‐3)'s brain demonstrating a small focus of PVNH adjacent to the right frontal horn. [Colour figure can be viewed at wileyonlinelibrary.com]

A 9‐year‐old male (see Figure 1, Family 1, III‐2) presented with global developmental delay, intellectual disability, attention‐deficit/hyperactivity disorder (ADHD), and prominent behavioural disturbances. The perinatal period was unremarkable; however, early infancy was complicated by recurrent bronchiolitis and accidental facial burns. Developmental milestones were delayed; walking was achieved at 18 months, with severely delayed speech. Dysmorphic features included coarse facies, broad hands, rocker‐bottom feet, hypoplastic toenails, and multiple café‐au‐lait macules. Behavioural comorbidities progressed to include oppositional defiant disorder and severe anxiety. Brain MRI demonstrated mild ventricular widening and prominent perivascular spaces, as well as a small focus of PVNH adjacent to the left frontal horn (see Figure 2). A singleton exome sequencing (ES) identified a heterozygous frameshift variant, *MAP1B*: c.4163_4164del (p.Glu1388Valfs*4), classified as pathogenic. This created premature translation‐termination codons (PTCs) and was expected to undergo nonsense‐mediated mRNA decay (NMD). His older sister (Figure 1, Family 1, III‐1) has mild intellectual disability, ADHD, anxiety, behavioural disturbances, and sleep issues. She was subsequently identified to have the same variant, and she is awaiting brain MRI. The variant was not found in their mother (II‐4), nor in two maternal half‐siblings (Figure 1, Family 1, III‐3, III‐6) with intellectual disability and behavioural disturbances. The proband's father (Figure 1, Family 1, II‐3) has not had formal cognitive testing but did not complete secondary school and has only basic literacy skills. He is not available for genetic testing or further medical evaluation due to incarceration.

### 2.2 Family 2

**FIGURE 3:**
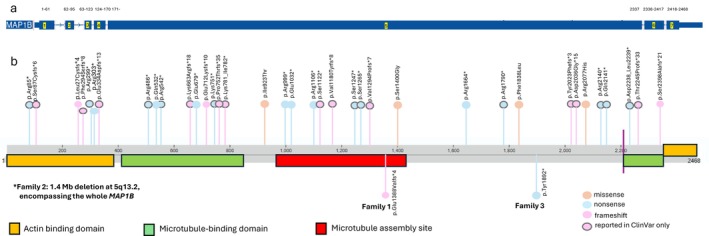
Disease‐causing variants of *MAP1B*, review of literature and ClinVar. (a) Schematic representation of *MAP1B* showing 7 exons with exon 5 contributing the majority of protein domains. Numbers above the exons represent the positions of amino acids in each exon. (b) Known published and ClinVar variants (above protein representation) and variants reported in this study (below). The schematic demonstrates active protein domains in relation to amino acid sequence. The vertical purple line represents a critical cleavage site that separates the heavy and light chains of the MAP1B protein, estimated to lie within 20 amino acids of residue 2200.

A 14‐year‐old male (Figure 1, Family 2, III‐4) was evaluated for moderate intellectual disability, autism spectrum disorder, ADHD, and behavioural dysregulation including anxiety and aggression. Birth at term following an uneventful pregnancy was normal, with appropriate anthropometrics and Apgar scores. His physical growth remained in the > 97th percentile for height and weight. MRI brain revealed PVNH around the frontal horns. Chromosomal microarray identified a *de novo* 1.4 Mb deletion at 5q13.2, encompassing the *MAP1B* (see Figure 3).

### 2.3 Family 3

The proband (Figure 1, Family 3, III‐1) was first assessed at 3 years of age for mild intellectual disability, focal epilepsy, behavioural disturbances, and significantly elevated BMI. A paternally inherited pathogenic *MAP1B* variant (c.5676T>A; p.Tyr1892*) was found on trio ES. This created a PTC and was expected to undergo NMD. Her MRI brain demonstrated high signal in the corona radiata but no evidence of PVNH. Her father (Figure 1, Family 3, II‐3) had a single seizure as a child, following which an MRI brain was performed at 14 years of age, which demonstrated a small focus of PVNH adjacent to the right frontal horn (see Figure 2). This p.Tyr1892* *MAP1B* variant was subsequently identified in two of the proband's paternal half‐siblings (Figure 1, Family 3, III‐5, III‐6). Both half‐siblings have global developmental delay requiring significant support and behavioural disturbances. III‐5's brain MRI showed foci of PVNH adjacent to the frontal horns. III‐6 has not had a brain MRI due to young age. III‐3 and III‐4 were not tested due to parental preference.

## 3 Discussion

Cortical development hinges on the precise spatial and temporal coordination of neuronal migration, axonal outgrowth, and synapse formation. This process relies heavily on cytoskeletal integrity, mediated by microtubules and actin filaments. MAP1B, one of the most abundant microtubule‐associated proteins in the developing brain, modulates microtubule dynamics, actin organization, and intracellular trafficking. The protein undergoes proteolytic cleavage to yield heavy and light chains, each exerting domain‐specific effects on cytoskeletal assembly. Phosphorylation by GSK3β and DYRK1A further refines its role in regulating microtubule stability and axon branching [5, 6].

Loss‐of‐function studies in *Map1b*‐deficient mouse models reveal pronounced deficits in axon elongation, dendritic arborisation, and synaptogenesis, consistent with the human phenotypes described herein. Notably, *MAP1B* has been shown to negatively regulate *KIF21A* activity, a kinesin motor protein involved in axon guidance, offering a mechanistic link to disorders such as congenital fibrosis of the extraocular muscles type 1 (CFEOM1), seen in patients with DCVs in both *MAP1B* and *KIF21A* [7, 8].

PVNH, a hallmark of disrupted neuronal migration, is increasingly recognized as a defining feature of *MAP1B*‐related disorders. Four of five described individuals with available neuroimaging demonstrated PVNH, with the exception being the Family 3 proband. This discrepancy raises important questions regarding penetrance and imaging sensitivity that warrant further investigation. Advanced imaging modalities, including diffusion tensor imaging (DTI) and connectome analysis, may provide deeper insights into subtle architectural disruptions in *MAP1B*‐related disease. Interestingly, no neuroimaging demonstrated polymicrogyria.

Unlike the typical posteriorly distributed PVNH seen in *FLNA* DCVs, *MAP1B*‐associated PVNH tends to localise anteriorly and symmetrically, frequently accompanied by polymicrogyria and corpus callosum dysgenesis. This distinctive neuroimaging phenotype likely reflects region‐specific vulnerabilities to disrupted cytoskeletal dynamics during early corticogenesis [9, 10, 11, 12]. Moreover, recent studies implicate *MAP1B* in axon initial segment (AIS) stability through its regulation of Na(v)1.6 (SCN8A) channel localisation, providing a plausible substrate for epileptogenesis in affected individuals [13, 14].

The clinical phenotypes observed across our cases are consistent with prior reports implicating *MAP1B* in a syndromic neurodevelopmental disorder characterized by global developmental delay, intellectual disability, behavioural disturbances (including autism and ADHD), and epilepsy [15]. Notably, despite distinct genotypes and inheritance patterns, including both *de novo* and familial truncating variants, all affected individuals displayed impairments in cognitive, motor, and social domains, suggesting a consistent effect of *MAP1B* haploinsufficiency on neurodevelopment. However, substantial intrafamilial and interfamilial variability was also evident, particularly in behavioural phenotypes, degree of cognitive impairment, epilepsy severity, and the absence of PVNH in the Family 3 proband. For example, the Family 3 father exhibits minimal functional impairment in adulthood despite a small focus of PVNH on brain MRI at age 14 years. Similarly, the Family 1 proband's sister (III‐1) has a much milder phenotype than her brother and may have gone undetected had she not been identified on familial variant testing. This variation may reflect modifying effects from genetic background, epigenetic factors, or compensatory mechanisms in cytoskeletal pathways.

A large Icelandic study identified the recurrent *MAP1B* variant p.(Glu712Lysfs*10) segregating with low IQ and non‐verbal learning disability, suggesting a broader cognitive profile linked to haploinsufficiency (10). A 966 kb deletion at 5q13.2, including *MAP1B*, was associated with intellectual disability and seizures, supporting its haploinsufficiency [16]. Furthermore, a *de novo* nonsense variant (p.Glu679*) and a familial frameshift variant (p.Ala2129Profs*107) were identified in children with PVNH, corpus callosum anomalies, and severe developmental phenotypes, consolidating the emerging genotype–phenotype correlation [11].

Beyond central nervous system phenotypes, *MAP1B* variants have been implicated in peripheral presentations. Missense variants in *MAP1B* have also been linked to autosomal dominant hearing loss in Chinese families. These findings expand the functional relevance of *MAP1B* to include cochlear development, likely mediated by its expression in spiral ganglion neurons [17].

Collectively, these observations position *MAP1B* as the second most frequently implicated gene in PVNH after *FLNA*, with variants identified in 4 of 196 PVNH cases across multiple cohorts.

*MAP1B* is 2459 amino acids long and consists of 7 exons, with exon 5 contributing the majority of protein domains [4]. Variants published to date appear to be concentrated more heavily around identified protein domains, as is the case with p.Glu1388Valfs*4 (our Family 1), although published variants have also been described elsewhere in exon 5. This proposed pattern is supported by variants reported as likely pathogenic or pathogenic in ClinVar (see Figure 3). In contrast, the previously unreported p.Tyr1892* (our Family 3) lies between known protein domains in a region with few other published *MAP1B* DCVs.

The phenotypic variability associated with *MAP1B* mutations remains striking. Factors such as variant type (e.g., missense vs. truncating), domain specificity, and modifying genetic or epigenetic influences likely contribute to this heterogeneity. Further functional studies and high‐resolution genotype–phenotype analyses will be essential for delineating pathogenic mechanisms and informing prognostic stratification.

From a clinical perspective, recognition of the *MAP1B*‐associated neuroimaging pattern characterized by anterior PVNH, insular polymicrogyria, and callosal thinning may provide a useful diagnostic clue in the evaluation of individuals with intellectual disability and epilepsy. Genetic testing panels for cortical malformations should routinely include *MAP1B* along with *FLNA* and *ARFGEF2*. Furthermore, longitudinal studies are needed to assess cognitive, behavioural, and seizure outcomes, especially in individuals with inherited variants and incomplete penetrance.

## 4 Conclusion

*MAP1B* is a pivotal cytoskeletal regulator essential for neuronal migration, axonal development, and synaptic architecture. DCVs in *MAP1B* are now recognized as a cause of a broad spectrum of neurodevelopmental disorders, including PVNH, intellectual disability, autism, epilepsy, and cortical malformations. It underscores the need for integrating molecular, imaging, and phenotypic data in the evaluation of neurodevelopmental disorders. Ongoing efforts to elucidate *MAP1B*'s role in cortical development and its interaction with other genetic regulators hold promise for future targeted therapeutic strategies.

## Ethics Statement

Ethical review and approval were not required for the publication of this case report in accordance with institutional and national guidelines. Written informed consent was obtained from the patient or legal guardian for publication of the clinical details and any accompanying images.

## Conflicts of Interest

The authors declare no conflicts of interest.

## Data Availability

The data that support the findings of this study are available on request from the corresponding author. The data are not publicly available due to privacy or ethical restrictions.
